# Integration of genetics and MRD to define low risk patients with B-cell precursor acute lymphoblastic leukaemia with intermediate MRD levels at the end of induction

**DOI:** 10.1038/s41375-024-02329-0

**Published:** 2024-07-05

**Authors:** Anthony V. Moorman, Amir Enshaei, Daniel Murdy, Melvin Joy, Judith M. Boer, Monique L. den Boer, Rob Pieters, Valerie de Haas, Martin A. Horstmann, Gabriele Escherich, Bertil Johansson, Hanne V. Marquart, Kjeld Schmiegelow, Jeremy Hancock, John Moppett, Mats Heyman

**Affiliations:** 1 Leukaemia Research Cytogenetics Group, Centre for Cancer, Translational and Clinical Research Institute, Newcastle University, Newcastle upon Tyne, UK; 2 Princess Máxima Center for Pediatric Oncology, Utrecht, The Netherlands; 3 Research Institute Children’s Cancer Center Hamburg, Hamburg and Department of Pediatric Oncology and Hematology, University Medical Center, 20246 Hamburg, Germany; 4 Department of Laboratory Medicine, Division of Clinical Genetics, Lund University, Lund, Sweden; 5 Department of Clinical Genetics and Pathology and Molecular Diagnostics, Laboratory Medicine, Region Skåne, Lund, Sweden; 6 Department of Clinical Immunology, Copenhagen University Hospital - Rigshospitalet, Copenhagen, Denmark; 7 Department of Clinical Medicine, Faculty of Health and Medical Sciences, University of Copenhagen, Copenhagen, Denmark; 8 Department of Paediatrics and Adolescent Medicine, University Hospital Rigshospitalet, Copenhagen, Denmark; 9 Bristol Genetics Laboratory, North Bristol NHS Trust, Bristol, UK; 10 Department of Haematology, Bristol Children’s Hospital, Bristol, UK; 11 Astrid Lindgren Children’s Hospital, Karolinska University Hospital and Department of Women’s and Children’s Health, Karolinska Institutet, Stockholm, Sweden

**Keywords:** Acute lymphocytic leukaemia, Risk factors

Successful treatment of acute lymphoblastic leukaemia (ALL) relies on assigning patients to appropriate risk groups. Modern protocols use a combination of age, white cell count (WCC), CNS involvement, immunophenotype, genetics and treatment response to assign patients to different treatment pathways which determines chemo-intensity, randomisations offered and eligibility for transplant or CAR-T. Measurable residual disease (MRD) at the end of induction (EOI) is recognised as the single most important risk factor in ALL. Patients with B-cell precursor (BCP) ALL and undetectable MRD at the EOI have an excellent outcome with risk of relapse (RR) and overall survival (OS) at 5 years of ~5% and ~99% respectively [1, 2]. At the other end of the spectrum, patients with EOI MRD ≥ 5% had a poor outcome with 5 year event free survival (EFS) < 70% and these patients are invariably defined and treated as high-risk [3–5]. Patients with high-risk genetics [*KMT2A* fusions, near-haploidy, low hypodiploidy, iAMP21, *TCF3::HLF*, and ABL-class fusions] or CNS disease at diagnosis also have an inferior outcome even when treated with intensive chemotherapy [6–9]. Collectively patients with excellent MRD response, high-risk or CNS disease account for roughly one-third of BCP-ALL patients and their risk classification is straight-forward. However, the risk classification of the remaining two-thirds of BCP-ALL patients with intermediate MRD levels (MRD-IR cohort) is debatable.

Analysis of UKALL2003 demonstrated that genotype-specific MRD thresholds improves the prediction of relapse and that the copy-number profiles of key genes was strongly linked with outcome [2, 10]. We sought to determine whether integrating MRD and genetics could identify clinically relevant subgroups within the MRD-IR cohort which might be suitable for treatment reduction or treatment intensification. Individual patient data were pooled from four studies whose 5 year survival rates were equal [11], namely UKALL2003 [12], NOPHO-ALL2008 [4], DCOG-ALL10^1^ and CoALL-07-03 [13] (Fig. S1). Each trial was approved by the relevant ethics committees and patients, parents or legal guardians gave written informed consent in accordance with the declaration of Helsinki. MRD was evaluated by PCR analysis of Ig/TCR rearrangements or, for NOPHO-ALL2008, flow cytometry to detect leukaemia-associated immunophenotypes. To examine MRD as a continuous variable, we log transformed the raw MRD value as previously described [2, 11]. Cytogenetics, fluorescence in-situ hybridisation or RT-PCR were used to detect relevant genetic abnormalities and classify patients into good, intermediate and high-risk groups (Fig. S1). Copy number data were derived from multiplex ligation-dependent probe amplification analysis using the P335/P202 SALSA kits (MRC Holland) and used to call the UKALL copy number alteration (CNA) profile and *IKZF1* deletions [10].

EFS, RR and OS were calculated using the Kaplan-Meier method. Relapses were defined as per original protocol. High-risk relapses comprised patients with a very early relapse ( < 18 months from diagnosis) or an early isolated BM relapse ( < 6 months off-treatment). We estimated the risk of relapse or death associated with individual risk factors using Cox regression analysis. Threshold analysis using the maxstat package in R was employed to determine the most discriminatory MRD threshold. ROC curves were used to assess the probabilities of the sensitivity and specificity ensuring sufficient discriminatory strength. All statistical analyses were performed using Intercooled Stata 16.0 or higher (Stata Corporation, USA), R version 3.4.3 or higher (http://www.R-project.org) and Python version 3.9.0 or higher (https://www.python.org/).

After a median follow-up time of 6.2 years, our IR cohort of 2,739 BCP-ALL patients, reflecting two-thirds of the total BCP-ALL population, had a 5-year RR, EFS and OS of 8.9% (95% CI 7.9–10.1%), 88.9% (87.6–90.1%) and 94.5% (93.5–95.3%) respectively. The patient characteristics of this cohort were: 54% male, 80% <10 years, and 61% NCI standard risk (Table S1). Increased levels of MRD at both the EOI and the end of consolidation (EOC) were associated with an elevated RR (Supplementary Table 2). However, models using EOI MRD were more predictive (as measured by Harrell’s C-index) confirming that EOI MRD was a better predictor of relapse than EOC MRD. Hence, the rest of our analyses focussed on integrating EOI MRD with genetics.

*ETV6::RUNX1* and high hyperdiploidy (HeH) status was available for most cases (2512/2739, 92%) with 758 (30%) being *ETV6::RUNX1* and 918 (37%) HeH (Supplementary Table 1). For both subtypes, we calculated the optimal MRD threshold for identifying patients with a low risk of relapse (Fig. S2). For *ETV6:RUNX1* the optimal threshold was <0.1% whereas for HeH it was <0.03% (Table 1). In each case, the specific threshold identified a large subset of patients with *ETV6::RUNX1* (84%) or HeH (60%) who had an outcome approaching that observed for patients with undetectable MRD at the EOI [1, 2] namely RR and OS rates of 4%, 98% and 5%, 97% respectively (Table 1). Importantly, patients with *ETV6::RUNX1* or HeH who had MRD above their specific thresholds had outcomes more similar to those reported for patients with intermediate risk genetics [12] namely RR rates of 13.8% and 11.2% respectively (Table 1).

**Table 1:** Frequency and outcome of patients (A) with different genetic abnormalities or profiles stratified by tailored MRD thresholds and (B) classified to the newly defined intermediate risk (IR) low and high groups.

	Optimum MRD threshold	Number of cases (%)	Risk of relapse at 5 years % (95% CI)	*P* value	EFS at 5 years % (95% CI)	*P* value	OS at 5 years % (95% CI)	*P* value
**A: Genetic Subgroup**								
* ETV6::RUNX1*	<0.1%	639 (84)	4.0 (2.7–6.0)	0.002	94.7 (92.6–96.3)	0.0004	98.3 (96.9–99.1)	0.0004
	≥ 0.1%	119 (16)	13.8 (8.2–22.6)		83.3 (74.3–89.3)		91.8 (84.2–95.9)	
High hyperdiploidy^a^	<0.03%	555 (60)	5.0 (3.4–7.3)	0.002	92.7 (90.2–94.7)	0.01	97.4 (95.6–98.4)	0.06
	≥ 0.03%	363 (40)	11.2 (8.0–15.2)		87.1 (82.9–90.4)		95.3 (92.4–97.2)	
UKALL-CNA good risk profile^b^	<0.05%	332 (77)	2.6 (1.3–5.2)	0.005	95.3 (92.3–97.1)	0.02	98.2 (96.0–99.2)	0.02
	≥ 0.05%	97 (23)	11.8 (6.7–20.3)		86.2 (77.5–91.8)		94.7 (87.7–97.8)	
UKALL-CNA poor risk profile^b^	<0.005%	131 (47)	4.2 (1.8–9.8)	0.001	92.9 (86.7–96.2)	0.003	95.1 (89.4–97.8)	0.02
	≥ 0.005%	150 (53)	16.3 (11.2–23.6)		80.3 (72.8–85.8)		89.2 (82.9–93.2)	
* IKZF1* deletion^c^	<0.01%	45 (34)	7.6 (2.5–21.9)	0.02	88.3 (74.0–95.0)	0.06	90.4 (76.1–96.3)	0.1
	≥ 0.01%	87 (87)	23.5 (15.7–34.5)		74.5 (63.6–82.6)		85.3 (75.5–91.4)	
**B: Proposed re-classification of MRD IR cohort**								
IR-low^d^	–	1291	4.3% (3.3–5.6)	<0.0001	94.0% (92.5–95.2)	<0.0001	98.0% (97.0–98.6)	<0.0001
IR-high^d^	–	926	14.4% (12.1–17.0)		82.9% (80.1–85.3)		90.8% (88.6–92.6)	

Notes: ^a^High hyperdiploidy (HeH) – 51–65 chromosomes.

^b^UKALL-CNA status was determined as detailed in Supplementary Fig. 1. Data were available for a subset of 710 cases including those with *ETV6::RUNX1* and HeH.

^c^*IKZF1* deletion status was available for 1008 cases including the 710 cases with UKALL-CNA status.

^d^IR-low = any case that has *ETV6::RUNX1* and EOI MRD < 0.1% (*n* = 639) or HeH and EOI MRD < 0.03% (*n* = 555) or UKALL-CNA good risk profile and EOI MRD < 0.05% (*n* = 97); IR-high= All remaining cases with *ETV6::RUNX1* (*n* = 119), HeH (*n* = 352) or a UKALL-CNA good risk profile (*n* = 31) were assigned to IR-high along with patients with a UKALL-CNA poor risk profile (*n* = 184) and B-other ALL (*n* = 235) or t(1;19) (*n* = 5) patients with EOI MRD ≥ 0.05%.

The distribution of key CNA can be used to classify cases according to the UKALL-CNA profile (Fig. S1) [10]. Threshold analysis revealed that patients with a good risk UKALL-CNA profile and EOI MRD < 0.05% had an excellent outcome (RR = 2.6%, OS = 98.2%) whilst those with MRD ≥ 0.05% had inferior outcomes (RR = 11.8%) (Table 1). The optimal threshold for patients with a poor risk UKALL-CNA profile was one log level lower at 0.005% but did not identify a prognostic favourable group like that obtained for the good risk UKALL-CNA profile. *IKZF1* deletions are associated with a poor outcome [14] and would be classified within the poor risk UKALL-CNA profile [10]. We examined the outcome of patients with an *IKZF1* deletion by MRD. Among 1008 patients tested, 132 (13%) cases were *IKZF1-*deleted (Fig. S1). Overall, patients with an *IKZF1* deletion had a poor outcome (RR = 18.2%, Table S1). Although the threshold analysis identified a discriminatory cutoff, it did not identify a group of patients with a very low RR or excellent OS (Table 1). CNA are secondary events which occur across primary subtypes. Future cohorts with better genomic characterisation will most likely provide better threshold based on primary events.

Collectively, these results support our previous assertion that the best way to integrate genetics and MRD to predict outcome is to define genotype specific thresholds [2]. Moreover, these findings indicate that within the MRD-IR cohort those patients with both good risk genetics (*ETV6::RUNX1*, HeH or UKALL-CNA good risk profile) and an individualised good MRD response at the EOI could define a subgroup with excellent outcome. Using the total MRD-IR cohort of 2739 cases, 2217 (81%) cases could be assigned to either IR-low or IR-high (Fig. S1). Cases fulfilling any of the following three criteria were IR-low: (1) *ETV6::RUNX1* and EOI MRD < 0.1% or (2) HeH and EOI MRD < 0.03% or (3) UKALL-CNA good risk profile and EOI MRD < 0.05%. Cases with *ETV6::RUNX1*, HeH or a UKALL-CNA good risk profile and MRD above these thresholds were assigned to IR-high along with patients with a UKALL-CNA poor risk profile as well as B-other\t(1;19) patients with EOI MRD ≥ 0.05% (Table S1).

**Fig. 1:**
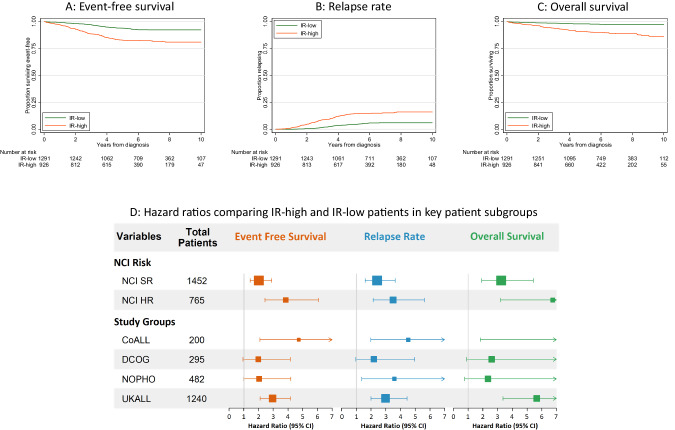
Outcome of patients classified as Intermediate Risk (IR) low and high according to genetics and end of induction MRD levels. **A–C** Kaplan-Meier plots illustrating the improved outcome for patients classified into the IR-low subgroup; **D** Forest plots showing the hazard ratios for EFS, RR, OS comparing IR-high against IR-low by NCI risk group and study group.

Patients classified as IR-high had a two to four-fold increased risk of relapse or death compared to the IR-low subgroup: hazard ratio for event = 2.77 (95% CI 2.12–3.61), *p* < 0.001; relapse = 3.05 (2.25–4.14), *p* < 0.001; death = 3.98 (2.66–5.93) (Table 1, Fig. 1A–C). Patients classified as IR-low had an outcome like that reported for patients with undetectable MRD at the EOI whereas patients classified as IR-high had outcomes consistent with that reported for patients with B-other ALL. Patients in the IR-low subgroup not only had a significantly lower RR but were also much less likely to have high-risk relapses compared to patients in the IR-high group. The ratios of very early, early, late relapses in the IR-low and IR-high subgroups were 5%/13%/83% v 21%/28%/53%, *p* < 0.001. Patients classified as IR-low were younger (*p* < 0.001) and were more likely to be NCI SR (*p* < 0.001) compared with IR-high patients but there was no difference in sex (*p* = 0.4) or WCC (*p* = 0.4) (Supplementary Table 1). Subgroup analyses revealed that the differential outcome between IR-low and IR-high was maintained across NCI risk groups and original cohorts (Fig. 1D). The outcome of those patients that could not be assigned to IR-low or IR-high due to missing data had an outcome close to the IR-high patients which is logical considering they were enriched for patients with B-other (60%) (Table S1).

The stratification of patients with intermediate risk MRD is crucial for designing effective treatment protocols including randomisations for treatment reduction. Our approach is novel in two respects. Firstly, it truly integrates MRD and genetics, the major risk factors, and secondly it focuses on identifying low risk rather than high-risk patients. This algorithm is simple, effective, and easy to implement based on routine diagnostic screening. We have not used newly identified genomic subtypes which could be viewed as a disadvantage. However, in terms of implementation, it is advantageous as only *ETV6::RUNX1*, HeH and the UKALL-CNA profile are needed. The first two abnormalities are screened for routinely whilst the UKALL-CNA profile can be assessed by SNP arrays which are now also used routinely [15]. This algorithm has been adopted by the ALLTogether1 trial (EudraCT: 2018-001795-38) to assign BCP-ALL MRD-IR patients to either IR-low for treatment reduction or IR-high for treatment intensification.

### Supplementary information

Supplementary Information
