# Supplementary material for: Integration of genetics and MRD to define low risk patients with B-cell precursor acute lymphoblastic leukaemia with intermediate MRD levels at the end of induction
Source: Leukemia. 2024 Jul 5;38(9):2023–6. doi: 10.1038/s41375-024-02329-0 (PMC11347368; doi:10.1038/s41375-024-02329-0)
Supplement: Supplementary file 1 — Supplementary Information [file 41375_2024_2329_MOESM1_ESM.pdf]

Supplementary Figure 1: CONSORT diagram illustrating the assembly of cohorts, exclusion criteria and the definition of each analysis dataset used in the study along with the frequency of the key variables. Individual patient data was pooled from UKALL2003, CoALL 07/03, DCOG-ALL10 and NOPHO-ALL2008 for cases patients who had detectable MRD but <5% at the end of induction.

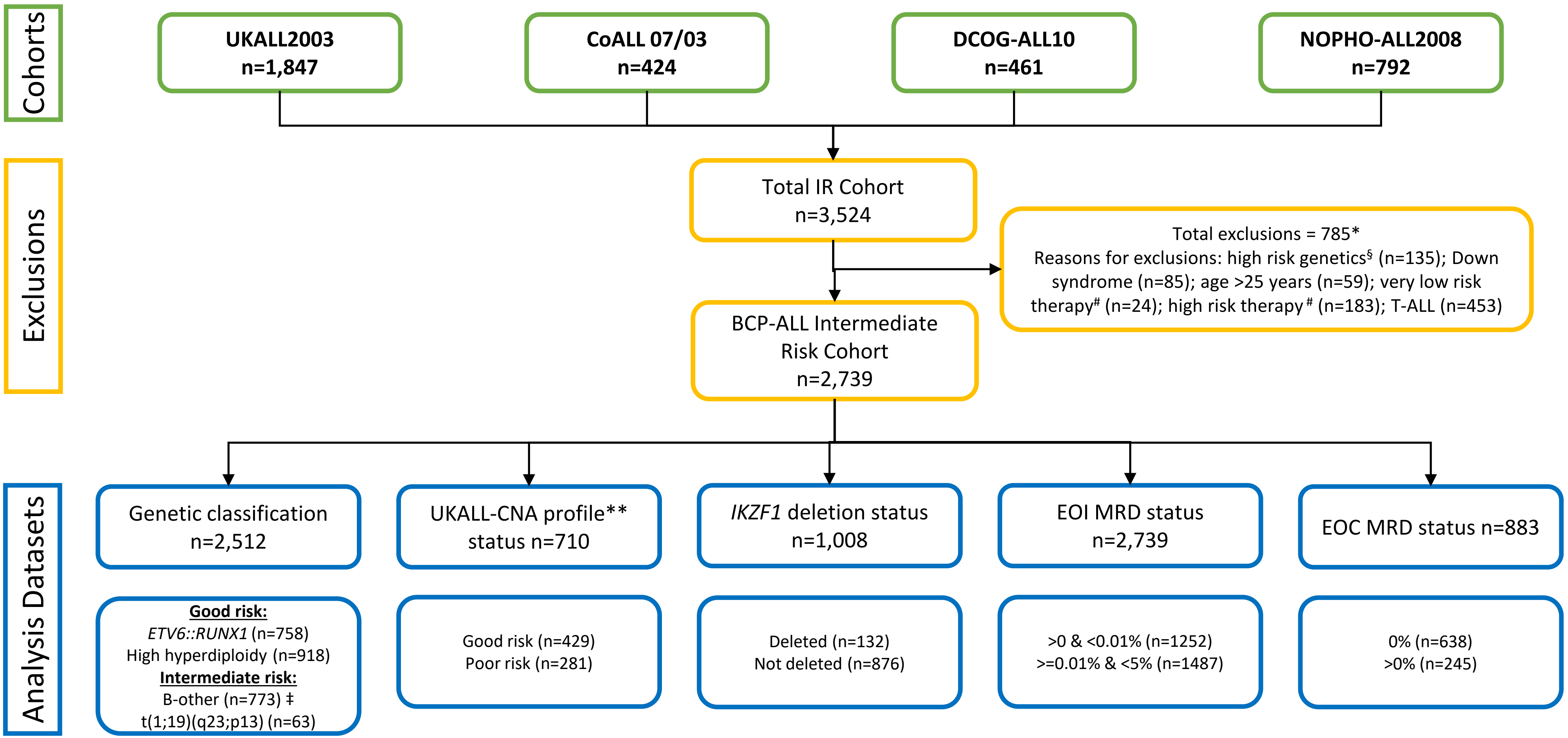

* Some patients had more than one of the exclusion criteria;

§ **High risk genetics**: *KMT2A* fusions, near-haploidy (<30 chromosomes), low hypodiploidy (30-39 chromosomes), intrachromosomal amplification of chromosome 21q (iAMP21) and t(17;19)(q23;p13) /*TCF3::HLF*, and ABL-class fusions (rearrangements involving *ABL1, ABL2, PDGFRB, CSF1R* fusions excluding *BCR::ABL1*);

\# Patients who received very low or high-risk therapy on CoALL98/03/09 or high-risk therapy on DCOG-ALL10 or NOPHO-ALL2008 were excluded from analysis because these treatment pathways were deemed significantly different across the four clinical trials.

** UKALL CNA good risk profile: Cases with (1) no deletions affecting *BTG1*, *CDKN2A/B*, *EBF1*, *ETV6*, *IKZF1*, *PAX5*, *RB1*, and PAR1; or (2) an isolated deletion of *BTG1* or *ETV6* or *PAX5*; or (3) the following deletion combinations: *ETV6* and *BTG1*, *ETV6* and *CDKN2A/B*, *ETV6* and a *PAX5* deletion. UKALL CNA poor risk profile: All other cases. Although this subset was generally representative of the whole cohort, these patients had a marginally lower RR at 5 years (7.1% (95% CI 5.3-9.3) v 9.6 (8.3-11.1), p=0.04).

‡ B-other cases had an abnormal karyotype or had tested negative for *ETV6::RUNX1*, high hyperdiploidy or *TCF3::PBX1*.

Supplementary Figure 2: Overview of the methodology used to select the optimal MRD thresholds for individual patient subgroups.

For each subgroup considered, cases were ordered according to the absolute MRD value. For each unique threshold, (A) the risk of relapse was calculated for patients with an MRD value below (black circles) and above (blue circles) that specific threshold (graph A). The greater the separation of the blue and black lines the greater discriminatory potential of the threshold; and (B) the size of the resulting subset was calculated (graph B). The threshold when the two lines cross indicates the mid-point of the cohort (i.e. the threshold that splits the cohort 50:50). Potential MRD thresholds are assessed by visually inspecting both plots (A & B) to determine the threshold with the greatest discriminatory potential subject to group size. For each potential optimal threshold, log rank tests looking for a difference in the overall survival distribution were calculated with the magnitude of test statistic assessed (graph C). To avoid inflation of Type I error's, the p-values were adjusted. Finally, ROC curves were used to assess sensitivity and specificity ensuring sufficient discriminatory strength (graph D).

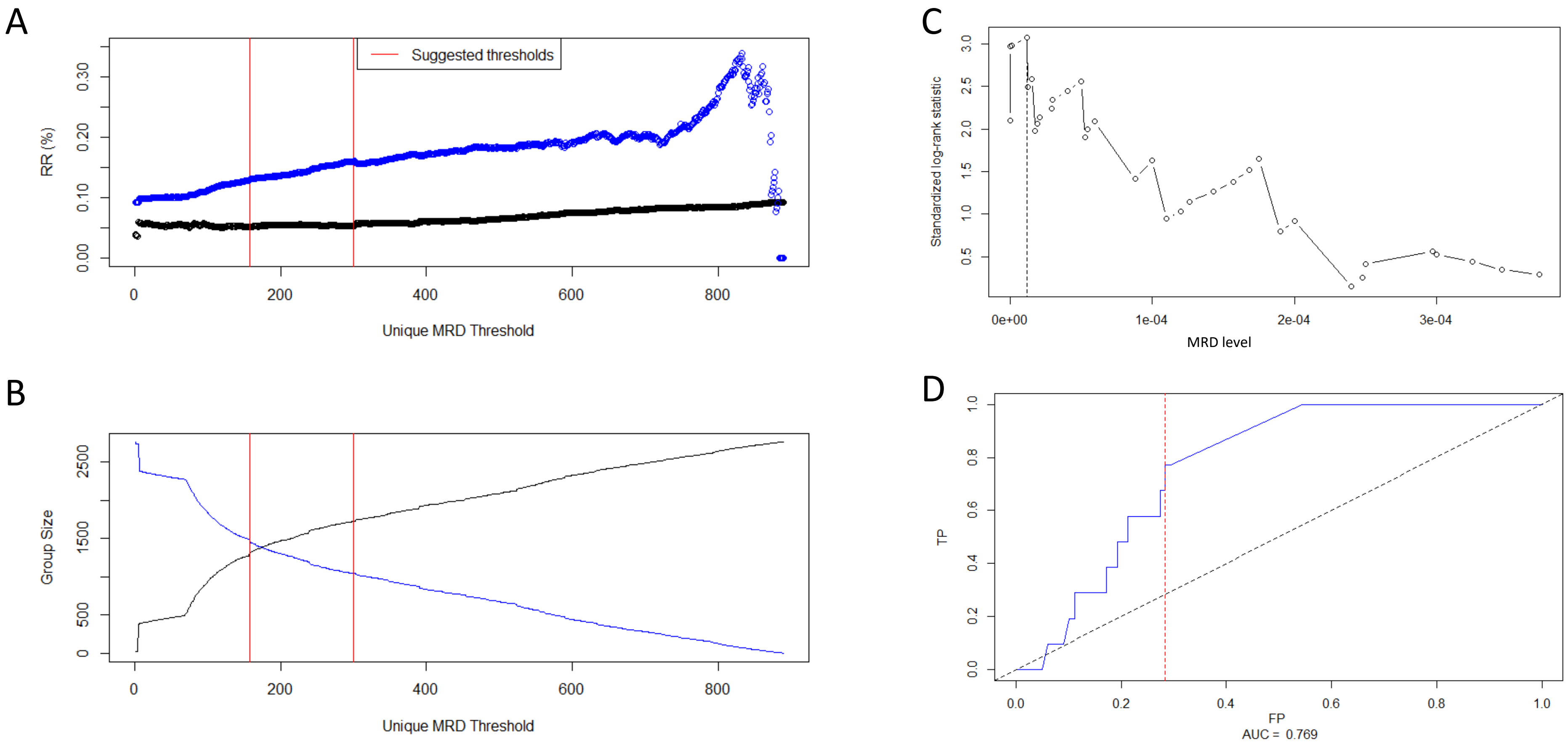

References: Lausen et al (1994) Computational Statistics, Heidelberg, Physica-Verlag, 483–496; Tripepi G et al (2009) Kindey International 76 (3):252-256; Hothorn T and Lausen B (2002) R News, 2(1):3–5.

Supplementary Table 1: Demographics, clinical features and outcome for 2,739 patients with BCP-ALL treated on UKALL2003, CoALL 07/03, DCOG-ALL10 and NOPHO-ALL2008 who had intermediate MRD levels at the end of induction stratified by genetic subtype, UKALL Copy Number Alteration (CNA) profile, and the two newly proposed subgroups.

	Total *	Primary Genetics Subtype				UKALL-CNA profile		*IKZF1* deleted	Proposed ALLTogether1 subgroup		
		High Hyperdiploidy	*ETV6::RUNX1*	*TCF3::PBX1*	B-other	Good risk	Poor risk		IR-low ‡	IR-high ‡	Unassigned ‡
Total	2,739	918	758	63	773	429	281	132	1,291	926	522
Sex, n (%) *											
Male	1,308 (54%)	437 (52%)	350 (53%)	30 (51%)	413 (57%)	227 (53%)	152 (54%)	67 (58%)	631 (54%)	443 (52%)	234 (56%)
Female	1,124 (46%)	406 (48%)	316 (47%)	29 (49%)	306 (43%)	202 (47%)	129 (46%)	48 (42%)	537 (46%)	406 (48%)	181 (44%)
Age (years), n (%)											
1-4	1,519 (55%)	568 (62%)	503 (66%)	36 (57%)	318 (41%)	267 (62%)	113 (40%)	50 (38%)	845 (65%)	426 (46%)	248 (48%)
5-9	676 (25%)	230 (25%)	193 (25%)	10 (16%)	175 (23%)	98 (23%)	89 (32%)	44 (33%)	315 (24%)	254 (27%)	107 (20%)
10-15	378 (14%)	85 (9%)	56 (7%)	13 (21%)	175 (23%)	49 (11%)	64 (23%)	30 (23%)	106 (8%)	161 (17%)	111 (21%)
16-24	166 (6%)	35 (4%)	6 (1%)	4 (6%)	105 (14%)	15 (3%)	15 (5%)	8 (6%)	25 (2%)	85 (9%)	56 (11%)
White blood cell count (x10^9/L), n (%) *											
<50	2,094 (86%)	782 (93%)	569 (86%)	43 (73%)	581 (81%)	373 (87%)	231 (83%)	94 (82%)	1029 (88%)	732 (87%)	333 (81%)
50-99	205 (8%)	43 (5%)	57 (9%)	11 (19%)	77 (11%)	34 (8%)	29 (10%)	11 (10%)	84 (7%)	75 (9%)	46 (11%)
>100	123 (5%)	15 (2%)	38 (6%)	5 (8%)	56 (8%)	21 (5%)	20 (7%)	9 (8%)	52 (4%)	38 (4%)	33 (8%)
NCI risk group *											
Standard	1,673 (61%)	676 (74%)	520 (69%)	31 (49%)	365 (47%)	316 (74%)	164 (58%)	65 (49%)	919 (71%)	533 (58%)	221 (42%)
High	1,066 (39%)	242 (26%)	238 (31%)	32 (51%)	408 (53%)	113 (26%)	117 (42%)	67 (51%)	372 (29%)	393 (42%)	301 (58%)
Outcome [%, (95% CI) at 5 years]											
Relapse Rate	8.9% (7.9-9.9)	7.3% (5.7-9.4)	5.4% (3.9-7.4)	11.4% (5.6-22.5)	12.7% (10-4-15.5)	4.7% (3.0-7.3)	10.8% (7.5-15.2)	18.2% (12.4-26.4)	4.3% (3.3-5.6)	14.4% (12.1-17.0)	11.5% (8.9-14.7)
Event Free Survival	88.9% (87.6-90.1)	90.6% (88.4-92.4)	93.0% (90.9-94.7)	85.7% (74.3-92.3)	85.1% (82.2-87.6)	93.2% (90.4-95.3)	86.1% (81.3-90.0)	79.2% (70.9-85.4)	94.0% (92.5-95.2)	82.9% (80.1-85.3)	86.3% (82.9-89.1)
Overall Survival	94.5% (93.5-95.3)	96.6% (95.2-97.6)	97.4% (95.9-98.3)	90.4% (79.8-95.6)	91.4% (89.0-93.2)	97.4% (95.3-98.5)	92.0% (88.0-94.6)	87.1% (79.8-91.9)	98.0% (97.0-98.6)	90.8% (88.6-92.6)	91.8% (89.0-94.0)

Notes: * Data were unavailable for some patients: sex n=307, white blood cell count n=317, genetics n=227. ‡ IR-low = any case that has *ETV6::RUNX1* and EOI MRD <0.1% (n=639) **or** HeH and EOI MRD<0.03% (n=555) **or** UKALL-CNA good risk profile and EOI MRD <0.05% (n=97); IR-high= Cases with *ETV6::RUNX1* (n=119), HeH (n=352) or a UKALL-CNA good risk profile (n=31) and EOI MRD above those thresholds along with patients with a UKALL-CNA poor risk profile (n=184) and B-other ALL (n=235) or t(1;19) (n=5) patients with EOI MRD ≥0.05%; Unassigned = patients with missing UKALL-CNA profiles status, missing genetic subtype or both; B-other is defined as all cases without good or high risk genetics (see Supplementary Figure 1).

Supplementary Table 2: Results of Cox models assessing the prognostic impact of different MRD variables across two different time-points

	Hazard Ratio for risk of relapse	95% CI	P	Harrell's C-index (%)	Log likelihood	Number of Cases
End of induction MRD						
log(MRD) [1]	1.21	1.15-1.27	<0.001	64.7%	-1834.58	2,739
τ(MRD) [2]	0.80	0.76-0.84	<0.001	64.7%	-1831.40	2,739
≥0.01% v <0.01%	2.45	1.85-3.24	<0.001	60.8%	-1843.45	2,739
End of consolidation MRD						
log(MRD) [1]	1.11	1.04-1.17	0.001	59.2%	-428.20	883
τ(MRD) [2]	0.83	0.74-0.92	0.001	56.3%	-428.34	883
>0% v 0% [3]	1.96	1.21-3.19	0.006	58.2%	-429.70	883

Notes: (1) log of MRD [zero values assigned a value of $1x10^{-6}$]. (2) τ(MRD) is a log transformed value [-ln(MRD)] of the absolute MRD level and indicates the risk of relapse associated with a log reduction in MRD. Prior to transformation, zero MRD values were assigned a value of $1x10^{-6}$ (one log below the minimum detection level of $1x10^{-5}$), MRD values $<1x10^{-5}$ were rounded up to $1x10^{-5}$ and MRD values ≥1 were rounded down to 0.99999; (3) Among 883 BCP-ALL cases with end of consolidation MRD, 638 (72%) had undetectable MRD (i.e. 0%);
